# First report of environmental *bla*_PAC-1_-carrying *Aeromonas enteropelogenes*

**DOI:** 10.1128/spectrum.01391-23

**Published:** 2023-11-01

**Authors:** Yang Zhong, Siyao Guo, Shuhua Thong, Joergen Schlundt, Andrea L. Kwa

**Affiliations:** 1 Department of Pharmacy, Singapore General Hospital, Singapore, Singapore; 2 Department of Clinical Translational Research, Singapore General Hospital, Singapore, Singapore; 3 School of Chemistry, Chemical Engineering and Biotechnology, Nanyang Technological University, Singapore, Singapore; 4 Emerging Infectious Diseases, Duke-NUS Medical School, Singapore, Singapore; 5 SingHealth Duke-NUS Medicine Academic Clinical Programme, Singapore, Singapore; Institut Pasteur, Paris, France

**Keywords:** bla_PAC-1_, *Aeromonas*, beta-lactamases, environmental microbiology, antimicrobial resistance, One Health

## Abstract

The *bla*_PAC-1_ has been reported in Central Asia and Europe countries like Afghanistan and France in *Aeromonas caviae* and *Pseudomonas aeruginosa* strains from animals and patients, respectively. However, there is no record of *bla*_PAC-1_-carrying strain from the natural environment, and *bla*_PAC-1_-carrying *Aeromonas* has not been reported in the Asia Pacific. Here, we report the first known enviromental *bla*_PAC-1_-carrying *Aeromonas enteropelogenes* in the world from reservoir water in Singapore. We have performed a comprehensive genetic environment alignment and comparison of *bla*_PAC-1_ between our strain and other strains from different countries and sources and found the *bla*_PAC-1_ located on a highly conserved gene cluster. We suggest that environmental *Aeromonas* strains may act as a hidden reservoir involved in the circulating of *bla*_PAC-1_. The finding of conserved *bla*_PAC-1_ cluster also suggested the existence of multiple transmission pathways of *bla*_PAC-1_ in the Asia-Pacific region, involving multiple sources and different species.

## LETTER

*Aeromonas* are opportunistic human pathogens, ubiquitous in waters, capable of transferring antimicrobial resistance genes (ARGs) with mobile genetic elements (1, 2). The *bla*_PAC-1_ (APM84516) was initially found in a clinical *Pseudomonas aeruginosa* strain AZPAE13864 from India in 2010 and identified as a novel class C β-lactamase (3). It was subsequently discovered in clinical multidrug-resistant (MDR) *P. aeruginosa* strains in Nepal (4), in France (from Mauritius or Afghanistan repatriated patients) (5), and in Singapore (6). When expressed in strain PAO1 (pUCP24*bla*_PAC-1_), *bla*_PAC-1_ confers resistance to ceftazidime (CAZ), cefepime (FEP), ceftolozane-tazobactam (CT), and ceftazidime-avibactam (CZA) (5). Despite *bla*_PAC-1_ being found in an *Aeromonas caviae* strain (PUTR01000038) from an Afghanistan dog (5, 7), the involvement of environmental bacteria with circulating *bla*_PAC-1_ remains unknown. Here, we report the first cephalosporin-resistant environmental *Aeromonas enteropelogenes* (LsrichE-8G) that harbors *bla*_PAC-1_.

LsrichE-8G (JALIHH000000000.1) was isolated from a water reservoir in Singapore in 2018 and confirmed as cephalosporin resistant using a microbroth dilution minimum inhibitory concentration (MIC) test (Table 1). Whole-genome sequencing (WGS) analysis revealed that it (genome size: 4,431,817 bp; GC content: 61.6%) belongs to a novel multilocus sequence type (MLST) of *A. enteropelogenes* species, highly similar (one allelic difference, *gyrB*_627*_novel) to ST1894. ResFinder 4.1 (default setting) identified that LsrichE-8G harbors *sul1* (sulfonamide), *dfrA1* (trimethoprim), *qacEΔ1* (disinfectant), and *bla*_MOX-12_ ARGs. The other two β-lactamase genes, *bla*_OXA-780_-like (p.K99Q, N100F) and *bla*_PAC-1_ (absent in ResFinder 4.1), were identified with CARD Resistance Gene Identifier (CARD RGI 6.0.1).

**TABLE 1:** Detailed information of strains included in genetic environment comparison^*a*^ ^,^ ^*b*^

Strain	Species	Accession	Collection year	Collection source	Collection country	Resistance (MIC µg/mL)
AFG_SD01_1510_Aca_091	*A. caviae*	PUTR01000038	2015	Dog	Afghanistan	FOX (S), CTX = 32, CTX + CLA = 16, CAZ = 8, CAZ + CLA = 1, FEP = 4, FEP + CLA = 1, ETP (S), MEM (S)
LSrichE-8G	*A. enteropelogenes*	JALIHH000000000.1	2018	Reservoir water	Singapore	CRO = 64, MEM ≤ 1, CEF > 16, CPD > 32, CIP ≤ 1,CTX = 64, CTX + CLA = 32, GEN ≤ 4, AMP > 16, CAZ = 32, CAZ + CLA = 16, CFZ > 16, IMI ≤ 0.5, TZP ≤ 4, FEP = 8, FOX = 8
PA0421	*P. aeruginosa*	SRR12669070	2015	Patient (wound)	Singapore	CT ≥ 128, CZA ≥ 128
PA0374	*P. aeruginosa*	SRR13396669	2015	Patient (wound)	Singapore	CT ≥ 128, CZA ≥ 128
PA0427	*P. aeruginosa*	SRR12669069	2015	Patient (wound)	Singapore	CT ≥ 128, CZA ≥ 128
PA1065	*P. aeruginosa*	SRR12669041	2018	Patient (blood)	Singapore	CT ≥ 128, CZA ≥ 128
PA1074	*P. aeruginosa*	SRR12669038	2018	Patient (blood)	Singapore	NR
PA174313	*P. aeruginosa*	MK534438.1	2017	Patient rectal	France (patient from Mauritius or Afghanistan)	TIC > 512, TZP = 128, CAZ > 128, FEP > 128, CT > 128, CZA > 128, ATM = 64, IPM = 1, MEM = 16, AMK > 128, TOB > 128, CIP = 16, FOS = 256, CST = 1
IOMTU487	*P. aeruginosa*	LC224309.1	2012–2013	Patient	Nepal	IPM = 2, MEM = 32, ATM = 128, CAZ = 512, AMK > 1,024, ABK > 1024, CIP = 32, CST ≤ 0.5
JUNP441	*P. aeruginosa*	DRR258736	2018–2020	Patient	Nepal	NR
JUNP415	*P. aeruginosa*	DRR258734	2018–2020	Patient	Nepal	NR
JUNP416	*P. aeruginosa*	DRR258735	2018–2020	Patient	Nepal	NR
JUNP507	*P. aeruginosa*	DRR258740	2018–2020	Patient	Nepal	NR

^*a*^ The basic genomic information was obtained from NCBI and relevant publications. The MICs of LSrichE-8G strain to different antibiotics were determined with microbroth dilution test performed with Sensititre ESBL Plate. The susceptibility of other strains was recorded according to their reports.

^*b*^ AMK, amikacin; AMP, ampicillin; CIP, ciprofloxacin; CST, colistin; FOS, fosfomycin; GEN, gentamicin; IPM, imipenem; MEM, meropenem; NR, not reported; TOB, tobramycin.

**Fig 1:**
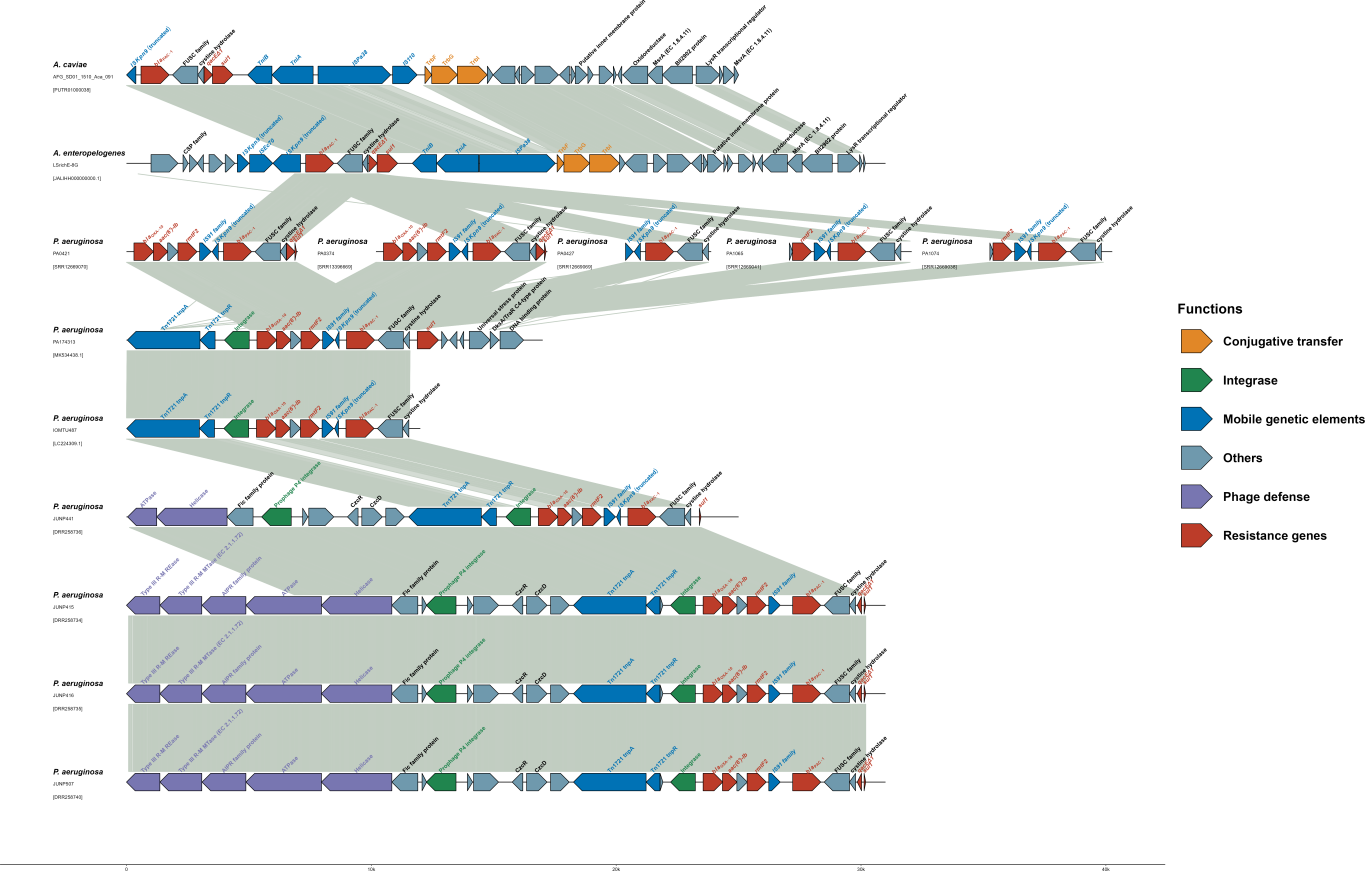
Genetic environment comparison of *bla*_PAC-1_ in *Aeromonas* strains and *P. aeruginosa* strains from different countries and sources. The details of bacterial strains selected for genetic environment comparison are listed in Table 1. The ARGs are labeled with red. Regions with 100% identity are linked with gray shading. The *bla*_PAC-1_ gene is found on a 40,033-bp contig in the *A. enteropelogenes* strain (LsrichE-8G, JALIHH000000000.1). The genetic environment of the contig is highly similar to the *bla*_PAC-1_ harboring contig of the *A. caviae* strain (accession: NZ_PUTR01000038.1). The *bla*_PAC-1_ gene in both *Aeromonas* and *P. aeruginosa* strains is located on the same gene cluster *ISKpn9*-PAC-1–2orfs*-qacEΔ1-sul1* regardless of countries and sources. The figure was plotted with the gggenomes R package (https://github.com/thackl/gggenomes).

In LsrichE-8G, *bla*_PAC-1_ was positioned on a 40,033-bp chromosomal contig, located in an *ISKpn9*-PAC-1–2orfs*-qacEΔ1-sul1* cluster (*ISKpn9* was intercepted by an *ISEc70*). *ISKpn9* in *Aeromonas* strains can insert into trap plasmid (8). Sequence alignments of *ISKpn9*-PAC-1–2orfs*-qacEΔ1-sul1* cluster, across strains from different species and sources (Table 1; Fig. 1) (5, 6), revealed that this cluster is highly conserved. In both *Aeromonas* strains, *ISKpn9*-PAC-1–2orfs*-qacEΔ1-sul1* cluster is followed by a pair of TniA/TniB putative transposases, an *ISPa38* (inserted by *IS110* in *A. caviae* (7)), and a set of conjugative transfer proteins TrbF/G/I. In *P. aeruginosa* strains, *ISKpn9*-PAC-1–2orfs*-qacEΔ1-sul1* cluster was consistently found downstream of a Tn3 family transposase (Tn1721-like), a Class 1 integron integrase (*IntI*), *rmtF2*, *aac(6′)-Ib*, and *bla*_OXA-10_ (5). However, rmtF*2*, *aac(6′)-Ib*, and *bla*_OXA-10_ were not gene cassettes as no *attI* or *attC* recombination sites were found (IntegronFinder 2.0). MDR Tn-1721-like transposon, which has similar GC content (59%) compared to *Aeromonas* genome (60%) than *P. aeruginosa* genome (66%), is likely formed through the joining of the *ISKpn9*-PAC-1–2orfs*-qacEΔ1-sul1* cluster mediated by *IS91* inserted in *ISKpn9* at 378 bp.

Cephalosporinases originated from environmental strains, like *bla*_CTX-M_ from environmental *Kluyvera* (9) and *bla*_CMY/MOX_ from *Aeromonas*, have spread to multiple human pathogens (10). From a “One Health” perspective, it is important to highlight the role of the aquatic environment as a hidden reservoir of ARGs. This study reflects the existence of multiple transmission pathways of *bla*_PAC-1_ across different sources (human, animal, and the environment) and different species (*P. aeruginosa* and *Aeromonas*). Despite low prevalence worldwide, *bla*_PAC-1_ is clinically important and warrants further investigation into its transmission strategy.

## Data Availability

The contigs of LSrichE-8G from this study have been deposited in the NCBI GenBank with accession number GCA_022869435.1.
